# Impact of particulate matter exposure on melanoma risk: A multicentre case–control study

**DOI:** 10.1111/jdv.20539

**Published:** 2025-01-17

**Authors:** Francesco Bellinato, Giovanni Adami, Maria Antonietta Reatini, Giorgio Cattani, Maria Vittoria Cannizzaro, Laura Del Regno, Ketty Peris, Paolo Gisondi, Giampiero Girolomoni

**Affiliations:** ^1^ Section of Dermatology and Venereology, Department of Medicine University of Verona Verona Italy; ^2^ Section of Rheumatology, Department of Medicine University of Verona Verona Italy; ^3^ Italian Institute for Environmental Protection and Research Rome Italy; ^4^ Dermatologia, Dipartimento Universitario di Medicina e Chirurgia Traslazionale Università Cattolica del Sacro Cuore Rome Italy; ^5^ Dermatologia, Dipartimento Scienze Mediche e Chirurgiche Fondazione Policlinico Universitario A. Gemelli IRCCS Rome Italy

## Abstract

**Background:**

The relationship between particulate matter (PM) exposure and melanoma risk remains largely unexplored. This study aims to investigate the association between PM10 and PM2.5 long‐term exposure and melanoma risk.

**Methods:**

Case–control study involving 2575 participants, comprising 1473 melanoma patients and 1102 healthy controls attending Departments of Dermatology of University Hospital in North and Central Italy. Demographic data, smoking status, history of sunburns and skin type were collected. PM10 and PM2.5 exposure levels were estimated for each participant's residential address using a Bayesian hierarchical model, providing daily concentrations at a 1 km^2^ spatial resolution from 2013 to 2021. Logistic regression analyses were performed to evaluate the association between PM exposure and melanoma risk, adjusting for potential confounders.

**Results:**

Melanoma patients and controls were 52% males and had a mean age of 63.89 and 61.66 years, respectively. The majority of melanoma patients had Fitzpatrick phototypes 2 (59%) and 3 (36%). There were no significant differences in the geographical distribution of cases and controls based on ZIP codes (*p* = 0.894). The average melanoma Breslow thickness was 1.01 mm, with 68.15% of cases diagnosed at stage 0 and IA. The multivariate logistic regression revealed a protective effect for higher PM10 (OR = 0.89, 95%CI: 0.86–0.92, *p* < 0.001) and PM2.5 levels (OR = 0.72, 95%CI: 0.68–0.76, *p* < 0.001). Darker skin phototypes (Fitzpatrick 4) and cigarette smoking were also associated with a reduced risk of melanoma.

**Conclusions:**

Higher levels of PM10 and PM2.5 may have a protective effect against melanoma, potentially due to the reduction in ultraviolet radiation exposure. Further research to understand the complex interactions between environmental factors and melanoma risk are needed.

## Graphical abstract

Long‐term exposure to higher levels of PM10 and PM2.5 may have a protective effect against melanoma, potentially due to the reduction in ultraviolet radiation.

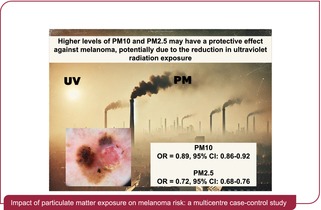

**Why was the study undertaken?**

- The potential impact of air pollutants exposure, including particulate matter (PM), on melanoma risk remains largely unexplored. Atmospheric pollution has a complex impact on the UV index, potentially reducing or increasing UV exposure depending on the composition and concentration of pollutants in the air.

**What does this study add?**

- In this case–control study, the multivariate logistic regression adjusting for potential confounders revealed a protective effect for higher PM10 (OR = 0.89, 95% CI: 0.86–0.92, *p* < 0.001) and PM2.5 levels (OR = 0.72, 95% CI: 0.68–0.76, *p* < 0.001). Darker skin phototypes (Fitzpatrick 4) and cigarette smoking were also associated with a reduced risk of melanoma.

**What are the implications of this study for disease understanding and/or clinical care?**

- Higher levels of PM10 and PM2.5 may have a protective effect against melanoma, potentially due to the reduction in ultraviolet radiation exposure and longitudinal studies with larger sample sizes are highly needed to elucidate the complex interplay between environmental exposures and melanoma risk.

## INTRODUCTION

Cutaneous melanoma has a rising trend in incidence rates over recent years.^1^ Incidence in Europe is about 25 cases per 100,000 population, six times as higher as it was 40 years ago^1^, ^2^, ^3^ and it is now the third most diagnosed cancer in Italy.^4^ Ultraviolet (UV) exposure is the most relevant well‐established environmental risk factor for melanoma, in particular, history of sunburn and use of tanning bed.^5^, ^6^ Acute intermittent exposure to UV radiation, primarily not only from sunlight but also from artificial sources like tanning beds, damages the DNA in skin cells, leading to mutations in genes that can initiate melanoma formation, that is, in p53 tumour suppressor gene.^7^

The progression to metastatic melanoma involves a complex process necessitating intricate interplay among external and internal triggers alongside tumour‐intrinsic and immune‐related elements.^8^ While the role of UV radiation in melanoma is widely recognized, the potential impact of other environmental factors, including particulate matter (PM) pollution, on melanoma risk remains largely unexplored.

PM is a mixture of solid particles and liquid droplets found in the air classified in PM10, that is, inhalable particles, with diameters that are generally 10 μm and smaller and PM2.5, fine inhalable particles, with diameters that are generally 2.5 μm and smaller. PM derives from various sources such as vehicle emissions, industrial activities and natural sources like wildfires. PM can penetrate the skin and potentially trigger inflammation and oxidative stress,^9^ which are potentially implicated in cancer development and progression. Pigmentary spots, premature photoaging, psoriasis and atopic dermatitis (AD) are inflammatory cutaneous diseases can be aggravated or exacerbated by exposure to air pollution.^10^, ^11^, ^12^, ^13^

On the other hand, PM can absorb, scatter or diffuse solar radiation and thus would attenuate the UV radiation from reaching the earth surface. The UV‐attenuating effect of PM depends on the density, composition and shape, which vary significantly with time and space.^14^ Hence, the specific association between PM exposure and melanoma risk remains elusive. The aim of this study was to evaluate the association between exposure to particulate matter and the risk of melanoma.

## MATERIALS AND METHODS

This case–control study included histopathologically confirmed melanomas consecutively recorded between 1 January 2022 and 31 December 2023 in the Department of Dermatology of the University Hospital of Verona and Policlinico Gemelli in Rome. Cases were compared with healthy controls who attended the clinics for nevi monitoring during the same period. Exclusion criteria for cases included a family history of melanoma and melanoma in the setting of genodermatoses, such as xeroderma pigmentosum. Controls were healthy individuals with no history of melanoma who were visited at the same clinic for nevus monitoring. Sociodemographic data, including age, sex, current or previous smoking habits, sun exposure, Fitzpatrick's skin phototype and residential address, were collected for both cases and controls. Detailed data on sun exposure were registered through structured interviews and questionnaires. Participants were asked about their average daily sun exposure (e.g. hours of sun exposure), use of sun protection (e.g. sunscreen, clothing), history of sunburns and outdoor activities. The residential address was used to determine the average daily exposure to coarse particulate matter (PM; 2.5–10.0 μm in diameter, PM10) and fine PM (<2.5 μm in diameter, PM2.5) from 1 January 2013 to 1 January 2021. To avoid potential biases, we verified the density of cases and controls within 10 km^2^ reference areas using geographic mapping software. This allowed us to ensure that cases and controls were similarly distributed across the study areas of Verona and Rome, minimizing the risk of geographic confounding. To avoid bias related to changes in residence, patients who had moved between the start and end of follow‐up were excluded.

PM10 mass concentrations at a 1 km^2^ spatial resolution and daily temporal resolution were estimated using a space–time statistical model, with the interpolation process relying on a Bayesian hierarchical approach. PM concentrations were calculated for each day by employing 11 independent spatial and spatio‐temporal explanatory variables alongside PM10 monitoring data. The PM10 hourly and daily average concentrations, collected over several years in the Italian monitoring network and archived in the InfoARIA database by ISPRA (Istituto Superiore per la protezione e la ricerca ambientale, https://www.isprambiente.gov.it/it), were determined using European reference or equivalent methods. These data underwent comprehensive validation in accordance with standard QA/QC procedures outlined in Directive 2008/50/EC. For subsequent analyses, the hourly data were aggregated on a daily basis to establish daily averages. The predictors used in this process included: (a) meteorological variables, known to strongly influence particulate concentration levels, (b) the Aerosol Optical Depth, a parameter representing the columnar optical thickness of aerosols, which is fundamental in evaluating the time and space variability of ground particulate matter concentrations, (c) elevation, (d) sand transport events, (e) Euclidean distance from main roads (used as a proxy for road traffic emissions) and (f) the impermeable surface, a key indicator of urbanization. The selection of these variables followed a priori knowledge of the phenomena involved, as previously described.^15^ To account for spatio‐temporal correlation, the model included a random effect, assumed to vary over time according to a first‐order autoregressive model with spatially correlated innovation. Additionally, a random effect, not spatially correlated, was modelled as an independent and identically distributed Gaussian process, capturing residual spatial variability at small scales. The capability of the Bayesian hierarchical model was assessed through a cross‐validation study following a well‐established procedure.^15^ The PM10 daily time series for each residential postcode associated with a patient were computed as the average of the model‐predicted values within the code coverage area. The average and cumulative (area under the curve—AUC) concentrations of PM10 and PM2.5 for each patient were calculated for the period 2013–2021 and 2020–2021, respectively. These values were then assigned to each participant based on their residential address, providing an estimate of their long‐term exposure to these air pollutants. Sensitivity analysis was conducted, excluding in situ melanoma and employing different thresholds of exposure, to assess the robustness of the findings.

### Statistical analysis

Descriptive statistics were used to summarize and characterize the features of the study population and variables. The primary analysis involved multivariable logistic regression to assess the association between PM exposure and melanoma risk. The dependent variable was the presence or absence of melanoma. Independent variables included PM10 and PM2.5 exposure, age, sex, smoking habits, sun exposure and Fitzpatrick skin phototype. The results were expressed as odds ratios (OR) with 95% confidence intervals (CI). All statistical analyses were conducted using Stata (version 18.0).

## RESULTS

**FIGURE 1:**
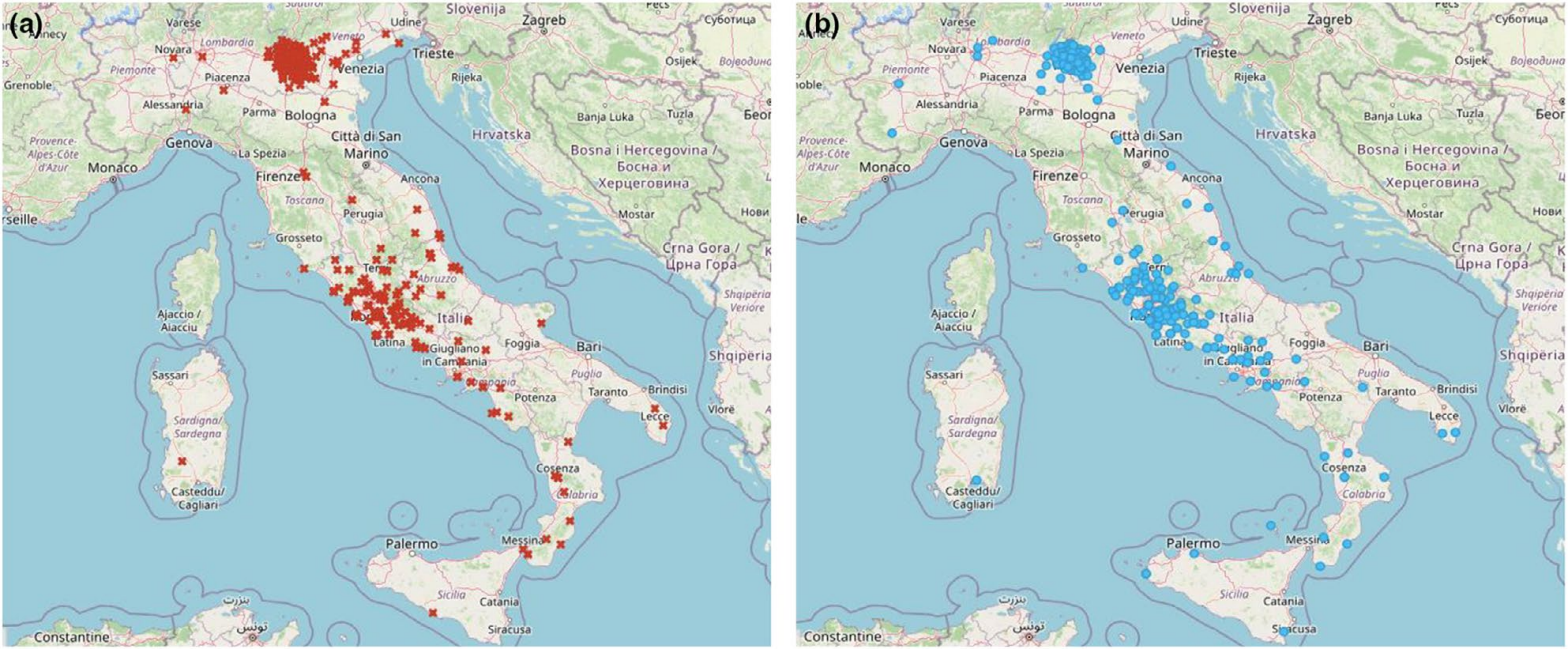
Geographical distribution of the cases (red cross) (a) and controls (blue dots) (b) included in the study.

The study included 2575 participants, comprising 1473 melanoma patients and 1102 healthy controls. The characteristics of the population are summarized in Table 1. Melanoma patients had a mean age of 63.89 years (SD = 15.15), 757 (51.59%) males, controls had a mean age of 61.66 years (SD = 18.54), 567 (51.45%) males. Geographical distribution also showed no significant disparity, particularly no significant differences between cases and controls in the ZIP codes of the addresses were found (*p* = 0.894; Figure 1).

**TABLE 1:** Demographic and clinical characteristics of the study population and mean PM levels and AUC exposure values in melanoma and controls from 2013 to 2021.

	Melanoma cases (*n* = 1473)	Healthy controls (*n* = 1102)	*p*
Age, mean ± SD years	63.89 ± 15.15	61.66 ± 18.54	0.665
Gender, male, *n* (%)	757 (51.59)	567 (51.45)	0.332
Cigarette smoking, *n* (%)	286 (43.27)	375 (56.73)	<0.001
History of sun burns, *n* (%)	1067 (76.38)	330 (23.62)	<0.001
Fitzpatrick phototype			
1	32 (2.17)	22 (2.00)	<0.001
2	864 (58.66)	461 (41.83)	
3	526 (35.71)	549 (49.82)	
4	51 (3.46)	68 (6.17)	
5	0 (0)	2 (0.18)	
6	0 (0)	0 (0)	
Breslow thickness (mm)	1.01 ± 1.71		
Stage*			
0 (in situ)	281 (20.86)		
IA	637 (47.29)		
IB	179 (13.29)		
IIA	69 (5.12)		
IIB	48 (3.56)		
IIC	19 (1.41)		
IIIA	42 (3.12)		
IIIB	31 (2.30)		
IIIC	24 (1.78)		
IIID	3 (0.22)		
IV	14 (1.04)		
PM10 (μg/m^3^)			
2021			
Mean	28.47 ± 5.98	27.39 ± 6.15	<0.001
AUC	10192.17 ± 2140.51	9820.02 ± 2207.38	<0.001
2020			
Mean	29.43 ± 4.57	28.64 ± 4.93	<0.001
AUC	10240.00 ± 1590.37	9980.37 ± 1734.06	<0.001
2019			
Mean	28.19 ± 4.50	27.45 ± 4.72	<0.001
AUC	10240.10 ± 1790.06	9893.57 ± 2093.81	<0.001
2018			
Mean	28.37 ± 4.26	27.71 ± 4.49	<0.001
AUC	10306.83 ± 1712.63	9985.27 ± 2034.85	<0.001
2017			
Mean	31.44 ± 6.04	30.29 ± 6.30	<0.001
AUC	11416.38 ± 2342.16	10917.11 ± 2640.21	<0.001
2016			
Mean	29.54 ± 4.57	28.89 ± 4.75	<0.001
AUC	10736.77 ± 1892.62	10429.18 ± 2165.07	<0.001
2015			
Mean	33.82 ± 5.82	32.85 ± 6.11	<0.001
AUC	12,261 ± 2347.68	11827.89 ± 2660.87	<0.001
2014			
Mean	29.27 ± 4.16	28.82 ± 4.36	0.0094
AUC	10617.99 ± 1713.18	10386.62 ± 2022.92	0.0018
2013			
Mean	32.20 ± 5.53	31.38 ± 5.83	0.0003
AUC	11678.83 ± 2218.71	11302.3 ± 2521.91	<0.001
PM2.5 (μg/m^3^)			
2021			
Mean	16.56 ± 3.2	15.80 ± 3.4	<0.001
AUC	5927.92 ± 1156.55	5664.05 ± 1241.65	<0.001
2020			
Mean	19.69 ± 5.3	18.67 ± 5.2	<0.001
AUC	6852.33 ± 1836.54	6507.54 ± 1926.43	<0.001

*Note*: According to the AJCC 8th.^32^

Abbreviations: AUC, Area under the curve; PM, particulate matter; SD, standard deviation.

A lower percentage of melanoma patients were cigarette smokers (43.27%) compared to healthy controls (56.73%) (*p* < 0.001). A significantly higher proportion of melanoma patients reported a history of sunburns (76.38%) compared to controls (23.62%) (*p* < 0.001). Regarding Fitzpatrick skin phototypes, the majority of melanoma patients had phototype 2 (58.66%) and phototype 3 (35.71%), with very few having phototype 1 (2.17%) and phototype 4 (3.46%). Phototypes 5 and 6 were nearly absent in both groups. The average Breslow thickness for melanoma cases was 1.01 mm. Staging of melanoma cases showed that 20.86% were in situ (stage 0), 47.29% were stage IA and the remaining were distributed across stages IB to IV, with the majority being in the early stages (IA and IB).

During the observation period, the patients were exposed to an average concentration of 29.69 (SD 4.80) μg/m^3^ PM10, and 17.75 (SD 4.04) μg/m^3^ PM2.5 (set limits PM10 40 μg/m^3^, PM2.5 25 μg/m^3^). Mean PM levels and AUC exposure values in melanoma and controls from 2013 to 2021 are summarized in Table 1.

The univariate logistic regression showed that both PM10 and PM2.5 levels were associated with an increased risk of melanoma. However, when adjusting for confounding factors, in the multivariate logistic regression, higher PM10 (OR = 0.89, 95% CI: 0.86–0.92, *p* < 0.001) and PM2.5 levels (OR = 0.72, 95% CI: 0.68–0.76, *p* < 0.001) were found to be protective against melanoma. Darker phototype (i.e. Fitzpatrick 4) was associated with a reduced risk of melanoma (OR = 0.32, 95% CI: 0.14–0.77, *p* = 0.010). Cigarette smoking was associated with reduced risk of melanoma in the univariate and multivariate models (OR = 0.28, 95% CI: 0.20–0.38, *p* = 0.010). Conversely, older age quartiles and history of sunburns were significant risk factors in the univariate and multivariate models (Table 2).

**TABLE 2:** Univariate and multivariate logistic regression assessing the risk of developing melanoma.

	Univariate logistic regression			Multivariate logistic regression		
	OR	95%CI	*p*	OR	95%CI	*p*
PM10 (continuous var)	1.03	1.02–1.05	0.001	0.89	0.86–0.92	0.001
PM25 (continuous var)	1.06	1.04–1.08	0.001	0.72	0.68–0.76	0.001
Fitzpatrick phototype						
1	Ref			Ref		
2	1.05	0.54–2.08	0.869	0.65	0.28–1.49	0.300
3	0.66	0.34–1.31	0.237	0.33	0.17–1.67	0.277
4	0.91	0.36–2.28	0.839	0.53	0.14–0.77	0.010
5						
Gender (male)	1.41	1.21–1.66	0.005	1.13	0.85–1.50	0.402
Age						
1st quartile	Ref			Ref		
2nd quartile	3.59	2.85–4.53	0.001	3.27	2.21–4.83	0.001
3rd quartile	4.68	3.70–5.93	0.001	4.24	2.83–6.37	0.001
4th quartile	6.77	5.30–8.66	0.001	6.44	4.22–9.84	0.001
Cigarette smoking	0.38	0.29–0.50	0.001	0.28	0.20–0.38	0.001
History of sun burns	6.65	5.17–8.55	0.001	8.50	6.38–11.59	0.001

Abbreviations: CI, confidence interval; OR, odds ratio.

## DISCUSSION

This study provides a pivotal analysis of the potential relationship between PM exposure and the risk of melanoma. The main result of this study suggests that increased exposure to PM10 and PM2.5 may have a protective effect against melanoma when considering multiple variables. Specifically, higher concentrations of PM10 (OR = 0.89, 95% CI: 0.86–0.92, *p* < 0.001) and PM2.5 (OR = 0.72, 95% CI: 0.68–0.76, *p* < 0.001) were associated with a decreased risk of developing melanoma. This inverse relationship was detected after adjusting for various confounding factors, including age, gender, smoking status, history of sunburns and Fitzpatrick skin phototype.

PM pollution exposure has been associated with flares of various inflammatory skin diseases such as atopic dermatitis, psoriasis and acne.^9^, ^10^, ^11^ PM can penetrate the skin barrier, leading to oxidative stress and inflammatory responses. Moreover, chronic exposure to high levels of air pollutants can worse premature skin ageing, hyperpigmentation and wrinkles.^16^

The detrimental effect on melanoma risk observed for PM10 and PM2.5 might be explained by their potential role in reducing exposure to UV radiation, the primary environmental risk factor for melanoma.^16^ Atmospheric pollution has a complex impact on the UV index, potentially reducing or increasing UV exposure depending on the composition and concentration of pollutants in the air. Some studies have reported that PM can reduce UV radiation by over 25%.^17^, ^18^ Maghrabi A et al. measured that both hourly and daily UVA and UVB values exhibit significant inverse correlations with PM10 concentrations, with hourly UVA values decreasing by 4 × 10^−3^ W/m^2^ and hourly UVB values decreasing by 2.7 × 10^−4^ W/m^2^ for every 1 μg/m^3^ increase in PM10. Similarly, daily UVA values decrease by 9 × 10^−3^ W/m^2^, and daily UVB values decrease by 11 × 10^−4^ W/m^2^ for every 1 μg/m^3^ increase in PM10.^14^, ^19^ It is unclear whether PM concentrations exceeded a critical threshold sufficient to create fog or significantly reduce UVR. A possible hypothesis is that critically elevated levels sustained over an extended period may have biological significance for melanoma risk, but further studies are needed to confirm it. In particular, the UV dose considered dangerous for increasing melanoma risk does not have a precise threshold, as the risk depends on several factors, such as exposure duration and intensity, phenotypic features and genetic predisposition.

Our study further confirmed other well‐known risk factors for melanoma, such as older age, history of sunburns and fair type.^7^ Conversely, individuals with Fitzpatrick phototype 4 had a reduced risk as previously reported.^20^

Interestingly, cigarette smoking was found to be associated with a reduced risk of melanoma in both univariate and multivariate models (OR = 0.26, 95% CI: 0.18–0.37, *p* = 0.010). Cigarette smoke is a complex mixture of reactive oxygen and nitrogen species, and aldehydes, closely resembling traffic‐ and industry‐derived air pollution.

Different studies have examined the impact of smoking on the risk of developing cutaneous melanoma, but the findings are mixed. Some of them, mostly prospective cohort studies, observed a negative association between cigarette smoking and melanoma risk,^21^, ^22^, ^23^, ^24^, ^25^, ^26^ while other studies did not find a significant association.^27^, ^28^ A meta‐analysis estimated the relative risk (RR) of melanoma to be 0.70 (95% CI: 0.63–0.78) for current smokers, RR = 0.90 (95% CI: 0.85–0.95) for former smokers and RR = 0.92 (95% CI: 0.87–0.94) for ever smokers.^29^ It was hypothesized that nicotine in cigarette smoke may shield the skin from UV‐induced inflammatory reactions, potentially lowering melanoma risk. Additionally, lifestyle differences, such as smokers potentially spending more time indoors, might be linked to a lower risk of melanoma among smokers.^30^ In line with our results, a recent case–control study by Sondermejier et al.^30^ detected a strong inverse relationship between cigarette smoking and melanoma risk in men. For females, the results were unclear and not statistically significant. These findings should be interpreted with caution because smoking has well‐established adverse health effects, and its potential protective role against melanoma might be attributed to complex biological interactions that warrant further investigation.

A recent study employing a two‐sample Mendelian randomization method to investigate the causal relationship between various air pollutants, including PM2.5, PM2.5–10, PM10 and nitrogen oxides, and the risk of cutaneous melanoma found no statistically significant association between air pollution and melanoma risk within European populations.^31^

Our study has some limitations, including the observational design, which limits our ability to establish causality between PM exposure and melanoma risk. Additionally, potential residual confounding factors, such as individual sun exposure behaviours and genetic predispositions, could not be fully accounted in the analysis. The exposure assessment relied on estimated PM concentrations based on residential addresses, which may not accurately reflect individual exposure levels, particularly for those who spend significant time outside their home area. The study also did not account for other environmental pollutants, lifestyle factors and several other uncontrolled confounders that could influence melanoma risk. Moreover, the cross‐sectional design only provides a snapshot in time and cannot capture long‐term exposure effects. We cannot rule out that certain patients may have relocated temporarily while maintaining the same official residence. Finally, the potential inaccuracies in self‐reported data on smoking, sunburn history and its recall bias, and skin phototype could introduce bias into our findings. The main strength of our study is that it encompasses a large cohort of 2575 participants, including 1473 melanoma patients and 1102 healthy controls, with detailed analysis of their demographic, environmental and clinical characteristics novel topic. Our findings are also novel given the paucity of data on this topic in the existing literature.

## CONCLUSIONS

Our findings suggest a neutral or even beneficial effect of exposure to higher PM10 and PM2.5 levels on melanoma risk. These results are preliminary and should be interpreted with caution because of many potential residual confounding. Longitudinal studies with larger sample sizes are highly needed to elucidate the complex interplay between environmental exposures and melanoma risk.

## AUTHOR CONTRIBUTIONS

Conceptualization: FB and GA; Formal Analysis: FB and GA; Resources: FB, GA, LDR, MAR and GC; Software: MAR and GC; Writing—original draft: FB; and Writing—review and editing: FB, GA, GG, PG and KP.

## FUNDING INFORMATION

This research was funded by the European Union—Next Generation EU—NRRP M6C2—Investment 2.1 Enhancement and strengthening of biomedical research in the NHS&quot.

## CONFLICT OF INTEREST STATEMENT

The authors declare that they have no known competing financial interests or personal relationships that could have appeared to influence the work reported in this paper.

## ETHICAL APPROVAL

The study was conducted in accordance with the Declaration of Helsinki, and the protocol was approved by the ethics committees of the participating institutions.

## ETHICS STATEMENT

Informed consent was obtained from all participants prior to their inclusion in the study (protocol number 1483).

## Data Availability

The data that support the findings of this study are available from the corresponding author upon reasonable request.
